# Study of a mechanism responsible for potential antidepressant activity of EMD 386088, a 5-HT_6_ partial agonist in rats

**DOI:** 10.1007/s00210-016-1245-3

**Published:** 2016-04-23

**Authors:** Magdalena Jastrzębska-Więsek, Agata Siwek, Anna Partyka, Lucyna Antkiewicz-Michaluk, Jerzy Michaluk, Irena Romańska, Marcin Kołaczkowski, Anna Wesołowska

**Affiliations:** Department of Clinical Pharmacy, Jagiellonian University Medical College, 9 Medyczna Street, 30-688 Cracow, Poland; Department of Pharmacobiology, Jagiellonian University Medical College, 9 Medyczna Street, 30-688 Krakow, Poland; Department of Neurochemistry, Institute of Pharmacology, Polish Academy of Sciences, 12 Smetna Street, 31-343 Krakow, Poland; Department of Pharmaceutical Chemistry, Jagiellonian University Medical College, 9 Medyczna Street, 30-688 Krakow, Poland; Adamed Ltd., Pieńków 149, 05-152 Czosnów, Poland

**Keywords:** Depression, 5-HT_6_ receptor agonist, EMD386088, Forced swim test, Rats, Monoamines metabolism

## Abstract

It was shown that 5-HT_6_ receptor agonists can exert pharmacological activity due to various modifications in monoamines’ level and metabolism activity in rats’ brain structures. This finding was correlated with antidepressant- or anxiolytic-like properties of these compounds. The study was designed to establish a possible mechanism of the antidepressant-like activity of the partial 5-HT_6_ receptor agonist EMD386088 (5-chloro-2-methyl-3-(1,2,3,6-tetrahydro-4-pyridinyl)-1*H*-indole hydrochloride) in rats. The concentrations of monoamines (dopamine (DA), noradrenaline (NA), and serotonin (5-HT)) and the rate of their metabolism were measured ex vivo in the brain structures (*hippocampus*, *nucleus accumbens*, *striatum*) using high-performance liquid chromatography (HPLC). The rats were killed after the forced swim test (FST); the collected tissue samples were used to ex vivo experiments. The potency of EMD386088 to blockade dopamine transporter (DAT) was tested in a functional in vitro study. FST was used to assess the involvement of D_1_- and D_2_-like receptor subfamilies in antidepressant-like properties of EMD386088. Neurochemical data from ex vivo experiments showed that antiimmobility activity of EMD386088 may be connected with the activation of dopaminergic system, while neither noradrenergic nor serotonergic ones are involved in its effect. EMD386088 also possesses a significant affinity for DAT which may be a mechanism in the abovementioned effect. Behavioral data seem to confirm the importance of dopaminergic system activation in antidepressant-like activity of EMD386088, since this effect, observed in the FST, was abolished by the preferential D_1_- and D_2_-like receptor subfamily antagonists SCH23390 and sulpiride, respectively. Dopaminergic system is involved in antidepressant-like activity of EMD386088.

## Introduction

A large amount of information has been collected in recent years on serotonin (5-hydroxytryptamine, 5-HT) 5-HT_6_ receptors and their possible physiological role within the central nervous system (CNS). The 5-HT_6_ receptor mRNA expression and receptor protein are largely confined to CNS, specifically striatum, nucleus accumbens (NAc), olfactory tubercle, cortex, and hippocampus (Kohen et al. 1996; Gérard et al. 1996, 1997; Roberts et al. 2002). Such specific localization in the CNS has suggested that ligands of this receptor may be involved in regulating mood changes (Monsma et al. 1993; Kohen et al. 2001; Wesołowska and Jastrzębska-Więsek 2011), thus the 5-HT_6_ receptor has emerged as an interesting molecular target for the development of drugs useful in mood disorders. Moreover, there is still an unresolved paradox that agonists/partial agonists and antagonists of this receptor evoke similar effects, i.e., procognitive (Fone 2008), antiobesity (Heal et al. 2008), anxiolytic, and antidepressant (Svenningsson et al. 2007; Wesołowska et al. 2007; Wesołowska 2008; Hirano et al. 2009; Carr et al. 2011; Nikiforuk et al. 2011; Jastrzębska-Więsek et al. 2014, 2015).

According to Gérard et al. (1997), 5-HT_6_ receptors are located outside 5-HT neurons, and are not autoreceptors. Ward et al. (1995) found that 5-HT_6_ receptor mRNA projection fields rather in regions of 5-HT-containing cell bodies, suggesting postsynaptic localization of these receptors. In vivo microdialysis studies that were carried out have revealed that both 5-HT_6_ receptor antagonists and agonists can modulate the levels of a variety of neurotransmitters in several brain regions associated with learning and memory and mood changes. A systemic administration of 5-HT_6_ receptor antagonists (e.g., SB 271046) has been shown to increase the release of acetylcholine and glutamate in the frontal cortex and dorsal hippocampus (Dawson et al. 2001; Riemer et al. 2003). In addition, the 5-HT_6_ receptor agonist, WAY-181,187, can elicit robust elevations in extracellular concentrations of γ-aminobutyric acid (GABA) in the dorsal hippocampus without affecting the release of noradrenaline (NA), 5-HT, dopamine (DA), or glutamate (Schechter et al. 2008). These data are consistent with the localization of 5-HT_6_ receptors in GABAergic interneurons suggested by colocalization of the receptor with the neuronal form of glutamic acid decarboxylase, GAD67, suggesting an indirect modulation of hippocampal glutamatergic neurotransmission via 5-HT_6_ receptor antagonist-mediated disinhibition (Ward and Dorsa 1996; Woolley et al. 2004). ST 1936, another 5-HT_6_ receptor agonist increased dialyzate DA and NA in the shell of NAc and in the medial prefrontal cortex (mPFX); it did not bind to the noradrenaline transporter (NET) and dopamine transporter (DAT) (Borsini et al. 2008; Valentini et al. 2011). As mentioned above, the scientific priority is to focus on finding information about these ligands and the differences in their effects/mechanisms of action. Studies conducted with EMD 386088, a 5-HT_6_ receptor partial agonist (Jastrzębska-Więsek et al. 2013) and a tool substance, fit well in this current research.

The systemic administration of EMD 386088, following acute and three times in 24-h treatment, caused an antidepressant-like effect detected during the modified forced swim test (FST) in rats. This effect of EMD 386088 was blocked by the selective 5-HT_6_ receptor antagonist SB 271046 administered in an inactive dose. Furthermore, its antidepressant-like effect, mediated directly by stimulation of 5-HT_6_ receptors, seems to be specific, since EMD 386088 did not affect rats’ locomotor activity, as measured in the open field (OF) apparatus. Moreover, the antidepressant-like activity of EMD 386088 was observed in an olfactory bulbectomy (OB) model of depression following chronic administration to rats (Jastrzębska-Więsek et al. 2015).

The present series of experiments was aimed at evaluating the mechanism of the antidepressant-like effect produced by EMD 386088 and observed in the modified FST in rats After the end of the FST, we carried out neurochemical ex vivo studies in rats’ brain structures (hippocampus, NAc, and striatum) to correlate the observed antidepressant effect of EMD 386088 with the levels of monoamines and their metabolites, the rate of monoamine metabolism, and indices of neural activity. Moreover, EMD 386088 was also tested in a functional in vitro assay of its potency to blockade DAT, the study conducted by Cerep company.

In the second part of experiments, the influence of D_1_- and D_2_-like receptor subfamily antagonists (SCH 23390 and sulpiride, respectively), on the antiimmobility effect induced by EMD 386088 has been examined in the FST. The dosage and time schedules of EMD 386088 were based on the results of our earlier studies (Jastrzębska-Więsek et al. 2015), whereas the antagonists of D_1_ and D_2_ receptor subfamilies were used at doses effective in blocking the effects induced by agonists of respective receptors (Rogóz and Skuza 2006; Hirano et al. 2007; Stuchlik et al. 2007).

## Materials and methods

### Animals

The experiments were performed on male Wistar rats (250–300 g) purchased from Charles River Laboratories (Germany). The animals were housed for a period of 6 days in polycarbonate Makrolon type 3 cages (dimensions 26.5 × 15 × 42 cm) in an environmentally controlled room (ambient temperature 21 ± 2 °C; relative humidity 50–60 %; 12:12 light/dark cycle, lights on at 8:00), in groups of four rats. Standard laboratory food (LSM-B) and filtered water were freely available. Animals were assigned randomly to treatment groups. All the experiments were performed by two observers unaware of the treatment applied between 9:00 and 14:00 on separate groups of animals. Procedures involving animals and their care were conducted in accordance with the current European Community and Polish legislation on animal experimentation. Additionally, all efforts were made to minimize animal suffering and to use only the number of animals necessary to produce reliable scientific data. The experimental protocols and procedures described in this manuscript were approved by the IV Local Ethics Commission in Warsaw (no 40/2008) and complied with the European Communities Council Directive of 24 November 1986 (86/609/EEC) and were in accordance with the 1996 NIH Guide for the Care and Use of Laboratory Animals.

### Drugs

The following drugs were used: 5-chloro-2-methyl-3-(1,2,3,6-tetrahydro-4-pyridinyl)-1*H*-indole hydrochloride (EMD 386088) was synthesized by Adamed (Pienków, Poland), SCH 23390 hydrochloride ((*R*)-(+)-7-chloro-8-hydroxy-3-methyl-1-phenyl-2,3,4,5-tetrahydro-1*H*-3-benzazepine hydrochloride, Tocris, UK). All drugs were dissolved in distilled water immediately before administration in a volume of 2 ml/kg. All the compounds were administered intraperitoneally (*i.p.*). EMD 386088 was given 30 min before the test, while the remaining compounds were injected 60 min before. Control animals received vehicle (0.9 % sodium chloride, NaCl) according to the same schedule.

### FST in rats

The experiment was carried out according to the modified, by Detke et al. (1995), method of Porsolt et al. (1978). On the first day of an experiment, the animals were gently individually placed in Plexiglas cylinders (40 cm high, 18 cm in diameter) containing 30 cm of water maintained at 23–25 °C for 15 min. On removal from water, the rats were placed for 30 min in a Plexiglas box under 60-W bulb to dry. On the following day (24 h later), the rats were replaced in the cylinder and the total duration of immobility, swimming, and climbing was recorded during the whole 5-min test period. The swimming behavior entailed active swimming motions, e.g., moving horizontally around in the cylinder. Climbing activity consisted of upward directed movements of the forepaws along the side of the swim chamber, and immobility was assigned when no additional activity was observed other than that necessary to keep the rat’s head above the water (Detke et al. 1995). Fresh water was used for each animal.

### Biochemical studies

#### Ex vivo: monoamine metabolism in rat brain structures

After the end of the FST, the rats were killed by decapitation and the brain was rapidly removed and dissected on different brain structures (*hippocampus*, *nucleus accumbens*, and *striatum*) on an ice-cold glass plate. The structures were frozen on solid CO_2_ (−70 °C) until used for biochemical assays. DA and its metabolites, the intraneuronal, 3,4-dihydroxyphenylacetic acid (DOPAC); the extraneuronal, 3-methoxytyramine (3-MT) and the final metabolite, homovanillic acid (HVA); NA and its main extraneuronal brain metabolite, normetanephrine (NM); and 5-HT and its intraneuronal metabolite 5-hydroxyindolacetic acid (5-HIAA) were assayed by means of high-performance liquid chromatography (HPLC) with electrochemical detection (ED). The tissue samples were weighted and homogenized in ice-cold 0.1 M trichloroacetic acid containing 0.05 mM ascorbic acid. After centrifugation (10,000×*g*, 5 min), the supernatants were filtered through RC58 0.2-μm cellulose membranes (Bioanalytical Systems, West Lafayette, IN, USA). The chromatograph HP 1050 (Hewlett-Packard, Golden, CO, USA) was equipped with Hypersil columns BDS-C18 (4 × 100 mm, 3 μm). The mobile phase consisted of 0.05 M citrate-phosphate buffer, pH 3.5; 0.1 mM EDTA; 1 mM sodium octyl sulfonate; and 3.5 % methanol. The flow rate was maintained at 1 ml/min. DA, NA, and 5-HT and their metabolites were quantified by peak area comparisons with standards run on the day of analysis (*ChemStation*, *Hewlett-Packard software computer program*).

#### In vitro: affinity for dopamine transporter (DAT)

EMD 386088 was tested in functional in vitro (human recombinant CHO cells) assay of its potency to block dopamine transporter (DAT) conducted by Cerep (Le Bois l’Eveque, 8600 Celle L’Evescault, France). The standard Cerep assay protocol was used (see for details: http://www.cerep.fr/cerep/users/pages/ProductsServices/BindingPlatform.asp, and http://www.cerep.fr/cerep/users/pages/catalog/Affiche_CondExp_Test.asp?test=52).

### OF test in rats

The experiment was performed in a darkened room using Motor Monitor System (Campden Instruments, Ltd., UK) consisted of two SmartFrame Open Field stations (40 × 40 × 38 cm) with 16 × 16 beams, located in sound-attenuating chambers and connected to a PC software by control chassis. Individual vehicle- or drug-injected animals were gently placed in the center of the station. An automated Motor Monitor System recorded ambulation (in X and Y axis), the number of rearing and peeping episodes, and total distance covered by a rat for 5 min.

### Statistical analysis

The data of behavioral studies were evaluated by an analysis of variance: one-way ANOVA (when one drug was given) or two-way ANOVA (when two drugs were used) followed by Duncan’s post hoc test; *p* < 0.05 was considered significant. The results of the biochemical studies were analyzed using one-way ANOVA followed by Duncan’s post hoc test when appropriate. The data were considered statistically significant when *p* < 0.05.

The total catabolism rate for DA was assessed from the ratio of the final DA metabolite concentration; HVA to DA concentration and expressed as the catabolic rate index [HVA] / [DA] × 100; the rate of DA monoamine oxidase (MAO)-dependent oxidation as the ratio [DOPAC] / [DA] × 100; the rate of DA COMT-dependent O-methylation as the ratio [3-MT] / [DA] × 100; the factor of DA reuptake inhibition as the ratio [3-MT] / [DOPAC] × 100. Analogously, the rate of NA metabolism was expressed as the ratio [NM] / [NA] × 100 and 5-HT as the ratio [5-HIAA] / [5-HT] × 100. The indices were calculated using concentrations from individual tissue samples (Antkiewicz-Michaluk et al. 2001).

## Results

### Antidepressant-like activity of EMD 386088 after single i.p. administration in the modified FST in rats

**Fig. 1:**
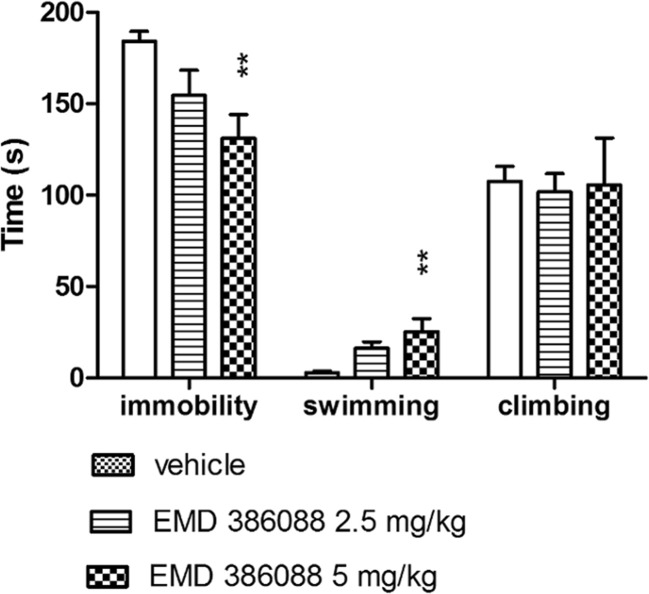
Antidepressant-like properties of EMD 386088 in the modified FST in rats. EMD 386088 was injected *i.p*. 30 min. Values represent the mean ± SEM during the 5-min test session; **p < 0.01, vs. vehicle group (one-way ANOVA is followed by the Duncan’s post hoc test); *N* = 7–8

EMD 386088 administered *i.p*. only at a dose of 5 mg/kg significantly decreased the immobility (ANOVA: F(2, 19) = 5.906; *p* < 0.01) and increased the swimming behaviors (ANOVA: F(2, 19) = 7.031; *p* < 0.01) of rats in the FST. There was no significant drug effect on the duration of climbing (ANOVA: F(2, 19) = 0.034; not significant, NS) (Fig 1).

### Biochemical studies

The ex vivo studies were conducted immediately after the end of the FST. The rats were killed by decapitation and the selected tissues were collected.

#### The effect of EMD 386088 on the concentration of DA and its metabolites and on the rate of DA metabolism in rat brain structures

The one-way ANOVA showed no significant effect of EMD 386088 (2.5 and 5 mg/kg) on DA and its extraneuronal metabolite 3-MT concentrations in the brain structures (Tables 1 and 2). At the same time, the statistical analysis revealed a significant effect of EMD 386088 on the level of the DA metabolites: an intraneuronal DOPAC and final HVA in NAc and striatum. The Duncan post-hoc test indicated that EMD 386088 (2.5 and 5.0 mg/kg) significantly decreased (from 20 to 30 %; *p* < 0.05) DOPAC and HVA concentration in a dose-related manner (Table 1).

**Table 1:** The effect of EMD 386088 on the concentration of DA and its metabolites in rat brain structures

Treatment	(mg/kg)	DA (ng/g t)	DOPAC (ng/g t)	3-MT (ng/g t)	HVA (ng/g t)
Hippocampus					
Vehicle	0	42 ± 10	9 ± 1.9	1.0 ± 0.5	14 ± 1.7
EMD 386088	2.5	58 ± 4	5 ± 0.6	1.5 ± 0.4	12 ± 0.9
	5	51 ± 4	5 ± 0.5	1.3 ± 0.6	13 ± 0.7
		F(2, 20) = 1.25 NS	F(2, 20) = 2.9 NS	F(2, 21) = 0.21 NS	F(2, 21) = 0.47 NS
Nucleus accumbens					
Vehicle	0	9756 ± 777	2066 ± 181	139 ± 18	1195 ± 102
EMD386088	2.5	9586 ± 389	1722 ± 73	169 ± 20	849 ± 41**
	5	10,017 ± 505	1614 ± 79*	143 ± 6	843 ± 39**
		F(2, 21) = 0.14 NS	F(2, 21) = 3.76 *p* < 0.05	F(2, 21) = 1.02 NS	F(2, 21) = 8.82 *p* < 0.01
Striatum					
Vehicle	0	11,386 ± 325	1773 ± 61	336 ± 7	1487 ± 111
EMD386088	2.5	11,305 ± 298	1589 ± 52*	329 ± 11	1228 ± 7 9*
	5	11,253 ± 327	1430 ± 47**	332 ± 12	1180 ± 66*
		F(2, 21) = 0.33 NS	F(2, 21) = 8.76 *p* < 0.01	F(2, 21) = 1.53 NS	F(2, 21) = 5.06 *p* < 0.05

EMD386088 was injected i.p. in a dose of 2.5 and 5 mg/kg; control group was treated i.p. with saline. The rats were decapitated about 30 min after drug injection. The results are expressed as the mean ± SEM (*N* = 8). The indices were calculated using concentrations from individual tissue samples. The concentration of dopamine and its metabolites were measured in ng/g tissue. The data were analyzed by means of one-way ANOVA, followed by Duncan’s post hoc test

*NS* not significant

Statistical significance: *p < 0.05, **p < 0.01 vs. respective control group

**Table 2:** The effect of EMD 386088 administration on rate of DA metabolism in rat brain structures

Treatment	(mg/kg)	[DOPAC]/[DA]	[3-MT]/[DA]	[3-MT]/[DOPAC]	[HVA]/[DA]
Hippocampus					
Vehicle	0	28 ± 5.1	1.7 ± 0.6	8 ± 2.8	56 ± 14.2
EMD386088	2.5	9 ± 1.2**	2.7 ± 0.9	27 ± 7.4*	22 ± 1.6*
	5	10 ± 1.0**	2.0 ± 1.3	23 ± 16.3	27 ± 2.1*
		F(2, 20) = 11.03 *p* < 0.001	F(2, 20) = 0.28 NS	F(2, 20) = 4.11 *p* < 0.05	F(2, 20) = 4.53 *p* < 0.01
Nucleus accumbens					
Vehicle	0	21 ± 0.7	1.4 ± 0.1	7 ± 0.3	12 ± 0.8
EMD386088	2.5	18 ± 0.4**	1.7 ± 0.2	10 ± 0.9.**	9 ± 0.4**
	5	16 ± 0.7**	1.4 ± 0.1	9 ± 0.1*	8 ± 0.5**
		F(2,21) = 18.3 *p* < 0.0001	F(2, 21) = 2.52 NS	F(2, 21) = 6.81 *p* < 0.01	F(2, 21) = 14.54 *p* < 0.0001
Striatum					
Vehicle	0	15 ± 0.3	2.9 ± 0.1	19 ± 0.6	13 ± 0.7
EMD386088	2.5	14 ± 0.4*	3.3 ± 0.1	23 ± 0.5**	10 ± 0.7*
	5	13 ± 0.4**	2.9 ± 0.1	23 ± 0.7**	10 ± 0.6*
		F(2, 21) = 13.99 *p* < 0.0001	F(2, 21) = 3.88 *p* < 0.05	F(2, 21) = 13.68 *p* < 0.0001	F(2, 21) = 4.35 *p* < 0.05

EMD386088 was injected i.p. in a dose of 2.5 and 5 mg/kg; control group was treated i.p. with saline. The rats were decapitated about 30 min after drug injection. The results are expressed as the mean ± SEM (*N* = 8). The rate of DA total metabolism was expressed as the ratio [HVA] / [DA] × 100; the rate of DA MAO-dependent oxidation as the ratio: [DOPAC] / [DA] × 100; the rate of the inhibition of dopamine reuptake as the ratio: [3-MT] / [DOPAC] × 100; and the rate of COMT-dependent O-methylation as the ratio: [3-MT] / [DA] × 100. The indices were calculated using concentrations from individual tissue samples. The data were analyzed by means of one-way ANOVA, followed by Duncan’s post hoc test

*NS* not significant

Statistical significance: *p < 0.05, **p < 0.01 vs. respective control group

The one-way ANOVA showed a significant effect of EMD 386088 administration on the rate of final DA metabolism, [HVA] / [DA] in the hippocampus (F(2, 20) = 4.53, *p* < 0.05), NAc (F(2, 21) = 14.54, *p* < 0.0001), and striatum (F(2, 21) = 4.35, *p* < 0.05). At the same time, the statistical analysis demonstrated a dose-dependent significant decrease in the rate of DA oxidation [DOPAC] / [DA] and no effect on the rate of DA methylation [3-MT]/[DA] after EMD 386088 (2.5 and 5 mg/kg) administration in all the investigated structures. The factor of dopamine reuptake inhibition expressed as the ratio [3-MT]/[DOPAC] was increased in the all investigated brain structures (Table 2).

#### The effect of EMD 386088 on the concentration of 5-HT and its metabolite and on the rate of 5-HT metabolism in rat brain structures

The one-way ANOVA showed no significant effect of EMD 386088 (2.5 and 5 mg/kg) administration on 5-HT and its intraneuronal metabolite 5-HIAA concentrations in all the investigated brain structures (Table 3).

**Table 3:** The effect of EMD 386088 administration on 5-HT and its metabolite concentration and 5-HT metabolism in rat brain structures

Treatment	(mg/kg)	5-HT (ng/g t)	5-HIAA (ng/g t)	[5-HIAA]/[5-HT]
Hippocampus				
Vehicle	0	275 ± 11	168 ± 15	61 ± 5
EMD386088	2.5	294 ± 11	185 ± 9	63 ± 2
	5	288 ± 11	173 ± 15	59 ± 4
		F(2, 21) = 0.79 NS	F(2, 21) = 0.44 NS	F(2, 21) = 0.22 NS
Nucleus accumbens				
Vehicle	0	481 ± 42	324 ± 21	69 ± 5
EMD386088	2.5	551 ± 16	354 ± 14	64 ± 3
	5	435 ± 31	298 ± 20	69 ± 3
		F(2, 21) = 3.44 NS	F(2, 21) = 2.17 NS	F(2, 21) = 0.41 NS
Striatum				
Vehicle	0	369 ± 19	358 ± 22	97 ± 4
EMD386088	2.5	381 ± 13	392 ± 16	103 ± 3
	5	346 ± 18	352 ± 18	103 ± 6
		F(2, 21) = 1.14 NS	F(2, 21) = 1.26 NS	F(2, 21) = 0.54 NS

EMD386088 was injected i.p. in a dose of 2.5 and 5 mg/kg; control group was treated i.p. with saline. The rats were decapitated about 30 min after drug injection. The results are expressed as the mean ± SEM (*N* = 8). The concentration of serotonin (5-HT) and its metabolite, 5-hydroxyindolacetic acid (5-HIAA), was expressed as ng/g tissue. The rate of 5-HT metabolism was expressed as the ratio [5-HIAA] / [5-HT] × 100. The indices were calculated using concentrations from individual tissue samples. The data were analyzed by means of one-way ANOVA, followed by Duncan’s post hoc test

*NS* not significant

#### The effect of EMD 386088 on the concentration of NA and its extraneuronal metabolite, normetanephrine, and on the rate of NA metabolism in rat brain structures

The one-way ANOVA demonstrated no significant effect of EMD 386088 (2.5 and 5 mg/kg) administration on NA and its metabolite NM as well as on the rate of NA metabolism in the hippocampus and NAc (Table 4).

**Table 4:** The effect of EMD 386088 administration on NA and its metabolite concentration and NA metabolism in rat brain structures

Treatment	(mg/kg)	NA (ng/g t)	NM (ng/g t)	[NM]/[NA]
Hippocampus				
Vehicle	0	312 ± 15	14 ± 1.3	5 ± 0.4
EMD386088	2.5	324 ± 16	16 ± 0.9	5 ± 0.3
	5	300 ± 22	15 ± 1.6	5 ± 0.4
		F(2, 21) = 0.46 NS	F(2, 21) = 0.45 NS	F(2, 21) = 0.31 NS
Nucleus accumbens				
Vehicle	0	443 ± 48	15 ± 1.0.8	4 ± 0.1
EMD386088	2.5	443 ± 40	14 ± 0.8	4 ± 0.2
	5	445 ± 47	13 ± 0.8	5 ± 0.1
		F(2, 21) = 0.01 NS	F(2, 21) = 0.0 NS	F(2, 21) = 0.99 NS

EMD386088 was injected i.p. in a dose of 2.5 and 5 mg/kg; control group was treated i.p. with saline. The rats were decapitated about 30 min after drug injection. The results are expressed as the mean ± SEM (*N* = 8). The concentration of NA and its metabolite, normetanephrine (NM), was expressed as ng/g tissue. The rate of NA metabolism was expressed as the ratio of the extraneuronal metabolite NM to NA: [NM] / [NA] × 100. The indices were calculated using concentrations from individual tissue samples. The data were analyzed by means of one-way ANOVA, followed by Duncan’s post hoc test

*NS* not significant

### The affinity of EMD 386088 for DAT

EMD 386088 showed significant affinity for human DAT (K_i_ = 41 nM). In the same experiment, K_i_ for the referenced compound 1-[1-(2-Benzo [*b*]thienyl)cyclohexyl)]piperidine (BTCP) was 6.5 nM.

### The influence of SCH 23390 and sulpiride on the antidepressant-like effect induced by EMD 386088 in the FST in rats

**Fig. 2:**
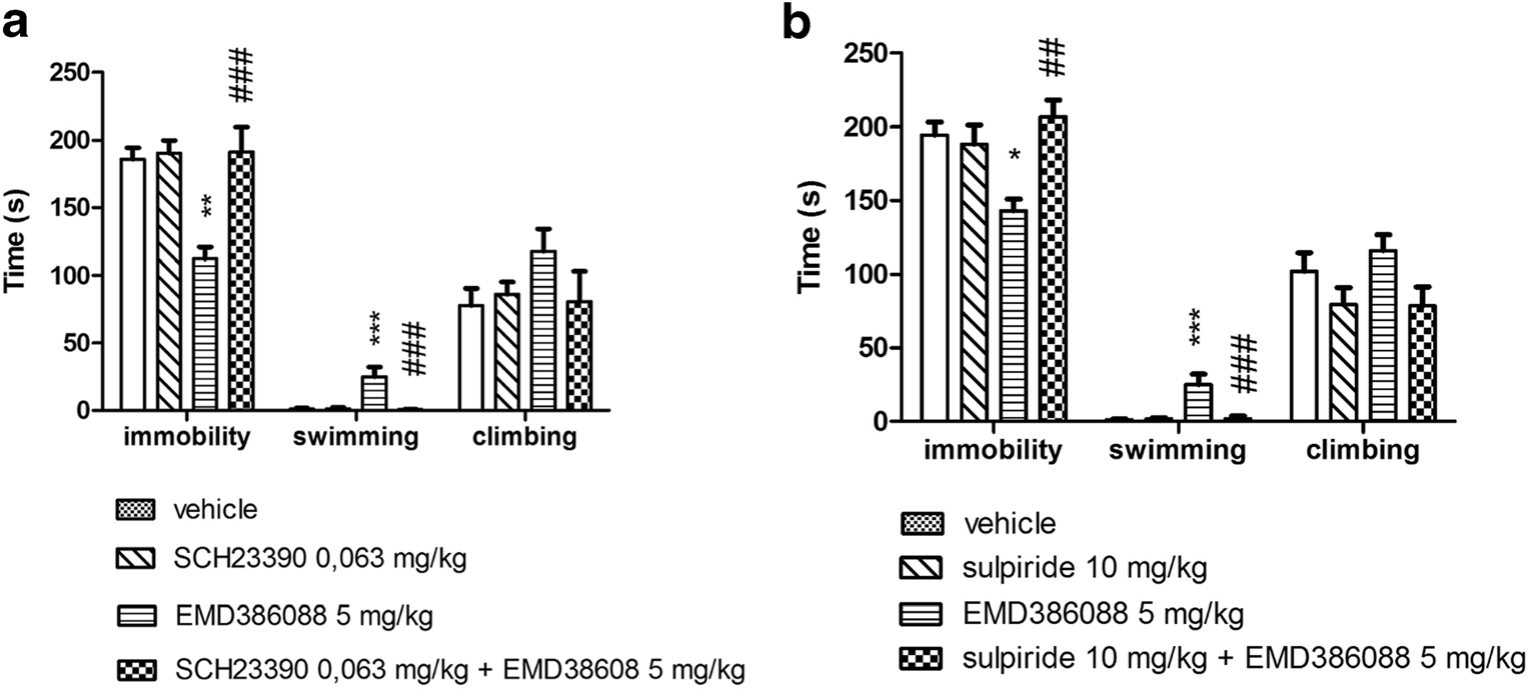
The influence of SCH 23390 (a) and sulpiride (b) on the antidepressant-like effect induced by EMD 386088 in the FST in rats. EMD 386088 was administered i.p. 30 min before the test, while SCH 23390 and sulpiride were given 60 min before. The animals were observed for 5 min. The results represent the mean ± SEM of 6–8 rats. The data were statistically evaluated by two-way ANOVA followed by Duncan’s post hoc test: *p < 0.05, **p < 0.01, ***p < 0.001 vs. respective vehicle group, ^##^ *p* < 0.01, ^###^ *p* < 0.001 vs. respective EMD 386088 group

EMD 386088 administered *i.p*. at a dose of 5 mg/kg showed antidepressant-like activity in FST, significantly decreasing the immobility and increasing the swimming behaviors of rats in this test. SCH 23390 (0.063 mg/kg) and sulpiride (10 mg/kg), administered alone did not change the behavioral parameters observed in FST (Fig. 2).

SCH 23390 (0.063 mg/kg) diminished an antidepressant-like effect of EMD 386088 (5 mg/kg) (Fig. 2a). The two-way ANOVA demonstrated a significant interaction of this cotreatment on the immobility time (F(1, 26) = 10.096, *p* < 0.01) and in the swimming behavior (F(1, 26) = 13.344, *p* < 0.01), but no significant interaction for climbing behavior was observed (F(1, 26) = 2.897, ns) (Fig.2a).

Sulpiride (10 mg/kg) given concomitantly with EMD 386088 (5 mg/kg) reversed the antiimmobility action evoked by EMD 386088 in the statistically significant manner (two-way ANOVA: F(1, 27) = 10.835, *p* < 0.01) as well as significantly decreased swimming behavior (two-way ANOVA: F(1, 27) = 12.803, *p* < 0.01) (Fig. 1b). The two-way ANOVA demonstrated no significant interaction for climbing behavior (F(1, 27) = 0.442, ns; Fig.2b).

#### OF test in rats

EMD 386088 (2.5 and 5 mg/kg) administered alone (Jastrzębska-Więsek et al. 2015) or in combination with SCH 23390 (0.063 mg/kg) or sulpiride (10 mg/kg) did not change the exploratory activity of rats (Table 5). SCH 23390 (0.063 mg/kg) given alone significantly decreased ambulation of rats or total distance and Y ambulation of rats, respectively. However, two-way ANOVA showed no significant interaction in all observed parameters of exploratory activity in those conducted experiments (Table 5).

**Table 5:** Effect of EMD 386088, SCH 23390, and sulpiride given alone or in combination on the rat locomotor activity measured in the OF test

Treatment	Dose (mg/kg)	Exploratory activity			
		Total distance (cm)	Rearings	Ambulation X	Ambulation Y
Vehicle + vehicle	0 + 0	2477 ± 164	65 ± 9	224 ± 28	236 ± 22
Vehicle + SCH 23390	0 + 0.063	1898 ± 196	55 ± 8	125 ± 13*	136 ± 13*
Vehicle + EMD 386088	0 + 5	3354 ± 358	86 ± 10	308 ± 29	330 ± 28*
SCH 23390 + EMD 386088	0.063 + 5	2227 ± 190	62 ± 5	172 ± 25	188 ± 22
		F(1, 23) = 1.4296; NS	F(1, 23) = 0.6977; NS	F(1, 23) = 0.5631; NS	F(1, 23) = 0.9170; NS
Vehicle + vehicle	0 + 0	2693 ± 222	84 ± 7	234 ± 26	237 ± 23
Vehicle + sulpiride	0 + 10	3324 ± 332	66 ± 9	352 ± 13	325 ± 35
Vehicle + EMD 386088	0 + 5	3354 ± 358	86 ± 10	308 ± 29	330 ± 28
Sulpiride + EMD 386088	5 + 10	3106 ± 358	81 ± 8	345 ± 32	357 ± 37
		F(1, 20) = 2.4700; NS	F(1, 20) = 0.5780; NS	F(1, 20) = 1.4950; NS	F(1, 20) = 0.9490; NS

EMD 386088 was injected i.p. 30 min, while the remaining compounds were injected 60 min before. Values represent the mean ± SEM during the 5-min test session compared to the respective vehicle + vehicle group (two-way ANOVA is followed by the Duncan’s post hoc test) (*N* = 6)

*NS* not significant

*p < 0.05

## Discussion

Our results indicate for the first time that the dopaminergic system is involved in the antidepressant-like activity of a 5-HT_6_ receptor partial agonist EMD 386088, which is observed in the modified FST in rats. In this study, we investigated the effects of *i.p.* treatment of EMD 386088 on behavioral (FST, OF test) and neurochemical parameters, and then we studied the effect of blockade of D_1_- and D_2_-like receptor subfamilies on the antidepressant-like properties of EMD 386088 in FST. The obtained behavioral results are in accordance with the biochemical ex vivo and radioligand in vitro investigations, which are presented in this paper.

In line with our earlier study (Jastrzębska-Więsek et al. 2015), the administration of EMD 386088 produced an antidepressant-like effect detected in the modified FST in rats. Specifically, EMD 386088, given at a dose of 5 mg/kg, exerts antidepressant-like properties as revealed by shortening of immobility and increasing in swimming behaviors (Fig 1). The effect of EMD 386088 was directly blocked by the selective 5-HT_6_ receptor antagonist SB-271,046 administered in an inactive dose (Jastrzębska-Więsek et al. 2015). Furthermore, its antidepressant-like effect, mediated by stimulation of 5-HT_6_ receptors, seems to be specific, because EMD 386088 did not affect rats’ total distance measured in the OF apparatus. The shortening of immobility time, induced by antidepressant drugs in FST, depends on the enhancement of the central 5-HT and catecholamine transmission (Porsolt et al. 1977, 1978; Borsini and Meli 1988; Borsini 1995).

To investigate the impact of EMD 386088 on the rate of monoamine (DA, NA, and 5-HT) metabolism, the biochemical ex vivo assays were conducted. For presentation neurochemical results, we selected three brain structures connected with monoaminergic function and with high 5-HT_6_ receptor mRNA expression, i.e., striatum, NAc, and hippocampus. Moreover, literature data has shown that modifications of monoamine transmission in these brain structures play an important role in the pathophysiology of depression. Therefore, NAc plays an important role in depression symptomatology, especially reducing motivation and causing anhedonia (Francis et al. 2015) as well as striatal dopamine modulates emotional and motor symptoms of depression (Rogers et al. 1998; Amsterdam and Newberg 2007). Neurochemical data showed that an observed antidepressant-like effect of EMD 386088 may be connected with the activation of monoaminergic, especially dopaminergic, system in rats’ brain. EMD 386088 given at the investigated doses (2.5 and 5 mg/kg) changed the DA metabolism and activity of the dopaminergic system in all investigated brain structures, i.e., hippocampus, NAc, and striatum. The administration of EMD 386088 did not change DA level and its extraneuronal metabolite 3-MT, but it significantly decreased the level of DA metabolites: an intraneuronal DOPAC and final HVA in the brain structures, except the hippocampus. Moreover, EMD 386088 significantly decreased the rate of final DA metabolism ([HVA]/[DA]) and the rate of DA intracellular oxidation ([DOPAC]/[DA]) of metabolic pathway. As it is well known, the intracellular DA oxidation by MAO is closely connected with the formation of free radicals leading to oxidative stress. Now there are many studies showing that depression is characterized by a significantly decreased antioxidant status as evidenced by lowered tryptophan, tyrosine, vitamin E, zinc, and reduced glutathione, which are all antioxidants (Maes 2008; Maes et al. 2011). In that light, antioxidant activity of EMD 386088 demonstrated by decreasing the rate of DA intracellular oxidation ([DOPAC]/[DA]) in all investigated brain structures correlates well with antidepressant-like activity of EMD 386088 in FST in rats. It may be concluded that intracellular inhibition of DA MAO-dependent oxidation would be one of the molecular mechanisms responsible for its antidepressant-like activity (Maes et al. 2011; Antkiewicz-Michaluk et al. 2014; Możdżeń et al. 2014). At the same time, the increase in a factor pointing to the DA uptake inhibition properties ([3-MT]/[DOPAC]) was observed. Such biochemical effects may suggest the stimulation of DA receptors through DA reuptake inhibition which occurred after EMD 386088 treatment. In fact, the affinity of EMD 386088 for DAT was also investigated. This compound binds to this transporter in nanomolar ratio of concentration, and this activity differs EMD 386088 from another 5-HT_6_ receptor agonist ST 1936 (Borsini et al. 2008). The DAT is the target for drugs used to treat depression (i.e., nomifensine), attention deficit hyperactivity disorder (ADHD) (i.e., methylphenidate), or addiction (bupropion) as well as for psychostimulants such as cocaine and amphetamine. Moreover, there was observed in DAT knockout mice frontal cortex significant decreasing of brain-derived neurotrophin factor (BDNF) mRNA levels (Haenisch and Bönisch 2011). The biochemical studies followed by Valentini et al. (2011) showed that systemic administration of ST 1936 dose-dependently increased dialyzate DA and NA in the shell of the NAc and mPFX. The mechanism of these effects was unlikely to be due to blockade of the DAT and of the NET, as ST 1936 did not bind to these transporters (Borsini et al. 2008; Valentini et al. 2011). Furthermore, this compound, in some behavioral tests, was shown to induce conditioned place preference and conditioned saccharin aversion and to be self-administered by rats, which indicate the DA-stimulant properties of ST 1936 in the NAc shell (Valentini et al. 2011). The study on effects of ST 1936 on electrical activity of putative mesencephalic dopaminergic neurons in the rat brain showed that it can either increase or decrease the basal firing rates of these DA-containing neurons in the ventral tegmental area and did not affect the basal firing activity of dopaminergic neurons in the substantia nigra pars compacta after systemic administration (Borsini et al. 2015). In case of the decrease in the basal firing rate, the authors suggested that the systemic administration of ST 1936 might have acted on different sites through the GABA neurons that control these DA neurons (Borsini et al. 2015). Previous studies have also shown that agonists of 5-HT_1A_ receptors are able to increase extracellular DA level in the PFX (Arborelius et al. 1993; Tanda et al. 1994). However, these agonists also decrease extracellular 5-HT through the activation of somatodendritic 5-HT_1A_ autoreceptors. In our investigations, EMD 386088, given in an active antidepressant-like dose (5 mg/kg), does not affect 5-HT and its intraneuronal metabolite (5-HIAA) concentrations in all the investigated brain structures. Moreover, its affinity for 5-HT_1A_ receptors is about 44-fold weaker (K_i_ = 320 nM) than that for 5-HT_6_ receptors (EC_50_ = 1 nM) (Mattsson et al. 2005). In this paper, we also have demonstrated that there was no significant effect of EMD 386088 administration on NA and its metabolite (NM) and on the rate of NA metabolism in the selected brain structures. Recently, in a microdialysis study, Valentini et al. (2011) demonstrated that systemic administration of ST 1936 dose-dependently increased dialyzate NA in the shell of NAc and in the mPFX, and to a lesser extent, in the NAc core. On the contrary, in the microdialysis study, the partial 5-HT_6_ receptor agonist WAY-181,187 following systemic acute *sc* administration modestly yet significantly decreased cortical 5-HT and DA levels and decreased striatal DA level (Schechter et al. 2008). The reported differences in effects evoked by 5-HT_6_ receptor agonists and partial agonists may be caused by various factors, i.e., their selectivity for 5-HT_6_ receptor over other receptors, the investigated brain structures, and the method used, i.e., microdialysis study vs. ex vivo study (WAY 181187 and ST 1936 vs. EMD 386088, respectively). The results obtained presently demonstrate that dopaminergic system, but not the noradrenergic one, plays an important role in the antiimmobility activity of EMD 386088, since this effect was abolished by the preferential D_1_- and D_2_-like receptor subfamily antagonists SCH 23390 and sulpiride, respectively. Both antagonists per se did not show any antidepressant-like effects. It is noteworthy that neither SCH 23390 nor sulpiride at the doses used, administered jointly with EMD 386088, modified the distance traveled by rats and measured in OF test; hence, their antagonism toward EMD 386088 in the FST cannot be attributed to a competing behavior, such as locomotor activity.

The results present in this paper seem to suggest a different indirect mechanism of antidepressant-like activity of a 5-HT_6_ receptor partial agonist EMD 386088 than that established for selective 5-HT_6_ antagonists. For example, SB 399885, the selective 5-HT_6_ receptor antagonist, exerts antidepressant-like properties by shortening immobility time in the FST in rats. Its activity in this model was connected with activation of D_1_- and D_2_-like receptors and α_2_-adrenoceptors, additionally not requiring any integrity of serotonergic neurons (Wesołowska 2007). SB 271046, another 5-HT_6_ receptor antagonist, significantly increased cortical and hippocampal extracellular concentrations of DA, NA (Lacroix et al. 2004), and acetylcholine in freely moving rats (Loiseau et al. 2008).

Also, we cannot exclude the role of other neurotransmitters in the antidepressant-like activity of EMD 386088 yet, i.e., acetylcholine. The clinical data suggest that hypercholinergic neurotransmission is associated with depressed mood stated and may be mediated through excessive neuronal nicotinic activation (Shytle et al. 2002). Moreover, in the preclinical investigations, it was shown that nicotinic receptor antagonists potentiate the antidepressant-like effects of imipramine and citalopram (Popik et al. 2003) and decrease the time of immobility in the forced swim and tail-suspension tests in mice (Borsini and Meli 1988; Cryan et al. 2005). Up to now, there is no in vivo or in vitro data available showing an effect of EMD 386088 on cholinergic system activity and acetylcholine ratio in brain structures, but such activity of EMD 386088 cannot be excluded, especially in view of the results demonstrating that 5-HT_6_ receptor antagonists increase extracellular acetylcholine level (Hirst et al. 2003, 2006) so probably 5-HT_6_ receptor agonists should it decreased, but till now, no data are available. On the contrary, it was also observed that both stimulation (by EMD 386088 and E-6801) and blockade (SB 271046) of 5-HT_6_ receptors exert procognitive action observed by reversing scopolamine-disrupted consolidation of passive avoidance task (Woods et al. 2012).

## Conclusions

The results described in this paper indicate that dopaminergic system is involved in the antidepressant-like activity of EMD 386088 via D_1_- and D_2_-like receptors and DAT. The intriguing neurochemical and receptor profile of EMD 386088, a partial 5-HT_6_ receptor agonist, supports the rationale for investigating its potential therapeutic benefits in the clinical management of depression and other disorders connected with hypofunction of the dopaminergic system.

## References

[CR1] Amsterdam JD, Newberg AB (2007). A preliminary study of dopamine transporter binding in bipolar and unipolar depressed patients and healthy controls. Neuropsychobiology.

[CR2] Antkiewicz-Michaluk L, Michaluk J, Mokrosz M (2001). Different action on dopamine catabolic pathways of two endogenous 1,2,3,4-tetrahydroisoquinolines with similar antidopaminergic properties. J Neurochem.

[CR3] Antkiewicz-Michaluk L, Wa̧sik A, Mozdzeń E (2014). Antidepressant-like effect of tetrahydroisoquinoline amines in the animal model of depressive disorder induced by repeated administration of a low dose of reserpine: behavioral and neurochemical studies in the rat. Neurotox Res.

[CR4] Arborelius L, Chergui K, Murase S (1993). The 5-HT1A receptor selective ligands, (R)-8-OH-DPAT and (S)-UH-301, differentially affect the activity of midbrain dopamine neurons. Naunyn Schmiedeberg's Arch Pharmacol.

[CR5] Borsini F (1995). Role of the serotonergic system in the forced swimming test. Neurosci Biobehav Rev.

[CR6] Borsini F, Meli A (1988). Is the forced swimming test a suitable model for revealing antidepressant activity?. Psychopharmacology.

[CR7] Borsini F, Stasi M, Minetti P (2008). Effect of ST 1936, a 5-HT6 ligand, on rodent adenylate cyclase and forced swimming test. Int J Neuropsychopharmacol.

[CR8] Borsini F, Bordi F, Poggi A, Di Matteo V (2015) Effects of ST1936, a selective serotonin-6 agonist, on electrical activity of putative mesencephalic dopaminergic neurons in the rat brain. doi:10.1177/0269881115573804 10.1177/0269881115573804 25735994

[CR9] Carr GV, Schechter LE, Lucki I (2011). Antidepressant and anxiolytic effects of selective 5-HT6 receptor agonists in rats. Psychopharmacology.

[CR10] Cryan JF, Valentino RJ, Lucki I (2005). Assessing substrates underlying the behavioral effects of antidepressants using the modified rat forced swimming test. Neurosci Biobehav Rev.

[CR11] Dawson LA, Nguyen HQ, Li P (2001). The 5-HT6 receptor antagonist SB-271046 selectively enhances excitatory neurotransmission in the rat frontal cortex and hippocampus. Neuropsychopharmacology.

[CR12] Detke MJ, Rickels M, Lucki I (1995). Active behaviors in the rat forced swimming test differentially produced by serotonergic and noradrenergic antidepressants. Psychopharmacology.

[CR13] Fone KCF (2008). An update on the role of the 5-hydroxytryptamine6 receptor in cognitive function. Neuropharmacology.

[CR14] Francis TC, Chandra R, Friend DM (2015). Priority communication nucleus accumbens medium spiny neuron subtypes mediate depression-related outcomes to social defeat stress. Biol Psychiatry.

[CR15] Gérard C, el Mestikawy S, Lebrand C (1996). Quantitative RT-PCR distribution of serotonin 5-HT6 receptor mRNA in the central nervous system of control or 5,7-dihydroxytryptamine-treated rats. Synapse.

[CR16] Gérard C, Martres MP, Lefèvre K (1997). Immune-localization of serotonin 5-HT6 receptor-like material in the rat central nervous system. Brain Res.

[CR17] Haenisch B, Bönisch H (2011). Depression and antidepressants: insights from knockout of dopamine, serotonin or noradrenaline re-uptake transporters. Pharmacol Ther.

[CR18] Heal DJ, Smith SL, Fisas A (2008). Selective 5-HT6 receptor ligands: progress in the development of a novel pharmacological approach to the treatment of obesity and related metabolic disorders. Pharmacol Ther.

[CR19] Hirano S, Miyata S, Onodera K, Kamei J (2007). Involvement of dopamine D1 receptors and a1-adrenoceptors in the antidepressant-like effect of chlorpheniramine in the mouse tail suspension test. Eur J Pharmacol.

[CR20] Hirano K, Piers TM, Searle KL (2009). Procognitive 5-HT6 antagonists in the rat forced swimming test: potential therapeutic utility in mood disorders associated with Alzheimer’s disease. Life Sci.

[CR21] Hirst WD, Abrahamsen B, Blaney FE (2003). Differences in the central nervous system distribution and pharmacology of the mouse 5-hydroxytryptamine-6 receptor compared with rat and human receptors investigated by radioligand binding, site-directed mutagenesis, and molecular modeling. Mol Pharmacol.

[CR22] Hirst WD, Rogers DC, Stean TO (2006). SB-399885 is a potent, selective 5-HT6 receptor antagonist with cognitive enhancing properties in aged rat water maze and novel object recognition models. Eur J Pharmacol.

[CR23] Jastrzębska-Więsek M, Siwek A, Kazek G (2013). Partial agonist efficacy of EMD386088, a 5-HT6 receptor ligand, in functional in vitro assays. Pharmacol Rep.

[CR24] Jastrzębska-Więsek M, Siwek A, Partyka A (2014). Pharmacological evaluation of the anxiolytic-like effects of EMD 386088, a partial 5-HT6 receptor agonist, in the rat elevated plus-maze and Vogel conflict tests. Neuropharmacology.

[CR25] Jastrzębska-Więsek M, Siwek A, Partyka A, et al. (2015) Antidepressant-like activity of EMD 386088, a 5-HT6 receptor partial agonist, following systemic acute and chronic administration to rats. Naunyn Schmiedeberg's Arch Pharmacol:2–11. doi:10.1007/s00210-015-1141-2 10.1007/s00210-015-1141-2 26077660

[CR26] Kohen R, Metcalf MA, Khan N (1996). Cloning, characterization, and chromosomal localization of a human 5-HT6 serotonin receptor. J Neurochem.

[CR27] Kohen R, Fashingbauer LA, Heidmann DEA (2001). Cloning of the mouse 5-HT6 serotonin receptor and mutagenesis studies of the third cytoplasmic loop. Mol Brain Res.

[CR28] Lacroix LP, Dawson LA, Hagan JJ, Heidbreder CA (2004). 5-HT6 receptor antagonist SB-271046 enhances extracellular levels of monoamines in the rat medial prefrontal cortex. Synapse.

[CR29] Loiseau F, Dekeyne A, Millan MJ (2008). Pro-cognitive effects of 5-HT6 receptor antagonists in the social recognition procedure in rats: implication of the frontal cortex. Psychopharmacology.

[CR30] Maes M (2008). The cytokine hypothesis of depression: inflammation, oxidative & nitrosative stress (IO&NS) and leaky gut as new targets for adjunctive treatments in depression. Neuro Endocrinol Lett.

[CR31] Maes M, Galecki P, Chang YS, Berk M (2011). A review on the oxidative and nitrosative stress (O&NS) pathways in major depression and their possible contribution to the (neuro)degenerative processes in that illness. Prog Neuro-Psychopharmacol Biol Psychiatry.

[CR32] Mattsson C, Sonesson C, Sandahl A (2005). 2-alkyl-3-(1,2,3,6-tetrahydropyridin-4-yl)-1H-indoles as novel 5-HT6 receptor agonists. Bioorg Med Chem Lett.

[CR33] Monsma FJ, Shen Y, Ward RP (1993). Cloning and expression of a novel serotonin receptor with high affinity for tricyclic psychotropic drugs. Mol Pharmacol.

[CR34] Możdżeń E, Papp M, Gruca P (2014). 1,2,3,4-tetrahydroisoquinoline produces an antidepressant-like effect in the forced swim test and chronic mild stress model of depression in the rat: neurochemical correlates. Eur J Pharmacol.

[CR35] Nikiforuk A, Kos T, Wesołowska A (2011). The 5-HT6 receptor agonist EMD 386088 produces antidepressant and anxiolytic effects in rats after intrahippocampal administration. Psychopharmacology.

[CR36] Popik P, Kozela E, Krawczyk M (2003). Nicotine and nicotinic receptor antagonists potentiate the antidepressant-like effects of imipramine and citalopram. Br J Pharmacol.

[CR37] Porsolt RD, Bertin A, Jalfre M (1977). Behavioral despair in mice: a primary screening test for antidepressants. Arch Int Pharmacodyn thérapie.

[CR38] Porsolt RD, Bertin A, Jalfre M (1978). “Behavioural despair” in rats and mice: strain differences and the effects of imipramine. Eur J Pharmacol.

[CR39] Riemer C, Borroni E, Levet-Trafit B (2003). Influence of the 5-HT6 receptor on acetylcholine release in the cortex: pharmacological characterization of 4-(2-bromo-6-pyrrolidin-1-ylpyridine-4-sulfonyl)phenylamine, a potent and selective 5-ht6 receptor antagonist. J Med Chem.

[CR40] Roberts JC, Reavill C, East SZ (2002). The distribution of 5-HT6 receptors in rat brain: an autoradiographic binding study using the radiolabelled 5-HT6 receptor antagonist [125I]SB-258585. Brain Res.

[CR41] Rogers MA, Bradshaw JL, Pantelis C, Phillips JG (1998). Frontostriatal deficits in unipolar major depression. Brain Res Bull.

[CR42] Rogóz Z, Skuza G (2006). Mechanism of synergistic action following co-treatment with pramipexole and fluoxetine or sertraline in the forced swimming test in rats. Pharmacol Rep.

[CR43] Schechter LE, Lin Q, Smith DL (2008). Neuropharmacological profile of novel and selective 5-HT6 receptor agonists: WAY-181187 and WAY-208466. Neuropsychopharmacology.

[CR44] Shytle RD, Silver AA, Lukas RJ (2002). Nicotinic acetylcholine receptors as targets for antidepressants. Mol Psychiatry.

[CR45] Stuchlik A, Rehakova L, Telensky P, Vales K (2007). Morris water maze learning in long-Evans rats is differentially affected by blockade of D1-like and D2-like dopamine receptors. Neurosci Lett.

[CR46] Svenningsson P, Tzavara ET, Qi H (2007). Biochemical and behavioral evidence for antidepressant-like effects of 5-HT6 receptor stimulation. J Neurosci.

[CR47] Tanda G, Carboni E, Frau R, Di Chiara G (1994). Increase of extracellular dopamine in the prefrontal cortex: a trait of drugs with antidepressant potential?. Psychopharmacology.

[CR48] Valentini V, Frau R, Bordi F (2011). A microdialysis study of ST1936, a novel 5-HT6 receptor agonist. Neuropharmacology.

[CR49] Ward RP, Dorsa DM (1996). Colocalization of serotonin receptor subtypes 5-HT2A, 5-HT2C, and 5-HT6 with neuropeptides in rat striatum. J Comp Neurol.

[CR50] Ward RP, Hamblin MW, Lachowicz JE (1995). Localization of serotonin subtype 6 receptor messenger RNA in the rat brain by in situ hybridization histochemistry. Neuroscience.

[CR51] Wesołowska A (2007). Study into a possible mechanism responsible for the antidepressant-like activity of the selective 5-HT6 receptor antagonist SB-399885 in rats. Pharmacol Rep.

[CR52] Wesołowska A (2008). The anxiolytic-like effect of the selective 5-HT6 receptor antagonist SB-399885: the impact of benzodiazepine receptors. Eur J Pharmacol.

[CR53] Wesołowska A, Jastrzębska-Więsek M (2011). Behavioral pharmacology: potential antidepressant and anxiolytic properties. Int Rev Neurobiol.

[CR54] Wesołowska A, Nikiforuk A, Stachowicz K (2007). Anxiolytic-like and antidepressant-like effects produced by the selective 5-HT6 receptor antagonist SB-258585 after intrahippocampal administration to rats. Behav Pharmacol.

[CR55] Woods S, Clarke NN, Layfield R, Fone KCF (2012). 5-HT6 receptor agonists and antagonists enhance learning and memory in a conditioned emotion response paradigm by modulation of cholinergic and glutamatergic mechanisms. Br J Pharmacol.

[CR56] Woolley ML, Marsden CA, Fone KCF (2004). 5-ht6 receptors. Curr Drug Targets CNS Neurol Disord.

